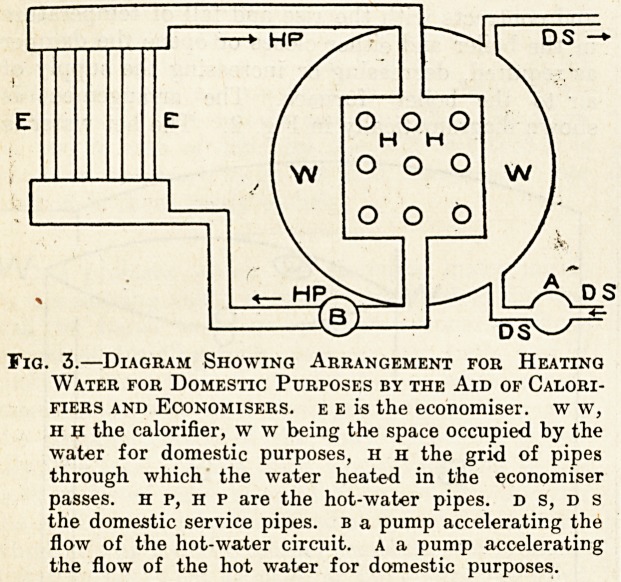# Bristol General Hospital

**Published:** 1916-06-24

**Authors:** 


### THE HEATING OF HOSPITALS.

**NOTE.—In a series of articles the writer proposes to describe the different systems of heating that are employed in hospitals. He will be pleased to answer any questions through "The Hospital," bearing upon the subject of heating or ventilation. Each system will be illustrated and described as exemplified at some hospital where it has been applied.**

### VIII. Bristol General Hospital.

### OPEN FIRES ASSISTED BY HOT-WATER RADIATORS, WITH ACCELERATED SERVICE FROM HOT-WATER BOILERS.

In the Bristol General Hospital three interesting methods of heating are employed. The new building is heated by hot-water radiators assisted by open fireplaces. Portions of the old building are heated in the same manner, and the remainder by hot air. The isolation ward is heated by hot-water radiators, heated by an anthracite stove in the basement. There are two floors in the isolation block with ten radiators, the anthracite stove which is employed for the purpose having the usual boiler attachment.

Fig. 1.—Diagram Showing Arrangement of Hot-water Radiators in the Isolation Ward, the Water being Heated in a Boiler attached to an Anthracite Stove. AS is the stove. B the boiler. RRR the radiators. HP, HP the hot-water pipe. CP the colder water return pipe.

Fig. 1 shows the arrangement. It burns one ton of coal in about ten days, and requires feeding only twice a day. The larger part of the old building is warmed by air heated on the old furnace system. The air which passes over the furnace is taken from a shaft opening into the middle of the yard, and is heated by a coke furnace with specially arranged flues to increase the heating surface. It passes over the smoke-box of the furnace, over the flues, and thence through ducts into the wards, corridors, etc. The hot air comes into the wards and corridors through gratings near the floor line; the exhaust air passing out through ducts near the ceiling, and thence to three towers provided for the purpose. The circulation of the air is due to the difference in the weight of the warm air passing up from the outlet ducts into the towers, and that of a similar column of the outside atmospheric air. The water for the radiators in the new building is heated in two Catena sectional boilers, each with two furnaces burning coke. The temperature to which the water in the boiler is raised is controlled by a damper in the boiler flue, which is operated by a rod attached to one end of a metallic bow fixed in the boiler shell. The bow expands and contracts with the rise and fall of temperature in the boiler and either closes or opens the damper as required, decreasing or increasing the supply of air to the boiler furnace. The arrangement is shown diagramatically in Fig. 2. The hot water is

Fig. 2.—Diagram Showing the Automatic Control of the Hot-water Boiler. B B is the boiler. F F the furnaces. D the damper. W the bow spring in contact with the boiler which raises and lowers the damper as the temperature of the water in the boiler falls or rises.

carried by a rising main to a ring main at the top of the building; thence it flows through the radiators and returns to a ring main in the basement.

There is the usual expansion tank in the upper part of the building to provide for the increase and decrease in volume of the water when hot and when cold. The circulation of the water through the pipes and radiators is "accelerated" by two reciprocating pumps connected in the return pipe.

#### The Steam-Boiler System and Hot-Water Supply.

The arrangement for heating the hot-water supply is very interesting, and, so far as the writer is aware, has not been used in any other hospital. There are two Lancashire boilers worked at 80-lb. pressure, and hand fired; one boiler is sufficient for the requirements of the hospital, the other being kept as spare. The hot gases from the boiler flues are made to heat water in two economisers; the hot water is used in two large reservoir calorifiers in a similar manner to that in which steam is employed for heating.* In every boiler furnace the heating is effected by the flow of the hot combustion gases, which are made to pass through flues arranged inside and outside of the boilers. When they are delivered to the chimney, if no "economiser" is employed they are still

FIG. 3.—DIAGRAM SHOWING ARRANGEMENT FOR HEATING WATER FOR DOMESTIC PURPOSES BY THE AID OF CALORIFIERS AND ECONOMISERS. E E is the economiser. W W, H H the calorifier, W W being the space occupied by the water for domestic purposes, H H the grid of pipes through which the water heated in the economiser passes. H P, H P are the hot-water pipes. D S, D S the domestic service pipes. B a pump accelerating the flow of the hot-water circuit. A a pump accelerating the flow of the hot water for domestic purposes.

at a very high temperature, seldom less than 600° F., and often at 800° and 900° F.; whereas 300° F. is sufficient to provide the necessary draught in the boiler furnaces. The economiser is a nest of vertical tubes through which water is kept circulating; the hot gases from the boiler flues flow over the outside surfaces of the pipes on their way to the chimney. The water heated in the economiser is usually employed for feeding the boiler, and it is of great importance it should not enter the economiser at a temperature lower than 90° F., as the gases flowing over cold pipes are stated to cause them to "sweat." Practically what happens is moisture is deposited from the hot gases themselves; the result is that the pipes rust; and, in addition to their being eaten away, the heating effect of the hot gases is not so good.

At the Bristol General Hospital the water is kept circulating through the economiser and through the heating coils in the calorifiers, so that the question of cold water entering the economiser does not arise. An electrically driven rotary pump is fixed in the circuit formed by the economiser and the calorifiers, and "accelerates" the flow of the heating water. Another electrically driven rotary pump accelerates the hot-water service, which is on the usual loop system. It will be noted that the calorifier itself is rendered more simple by this arrangement than with heating by steam, for there is no condensed steam to provide for. Fig. 3 shows the arrangement of the calorifiers, economisers, and the pumps. In addition to the hot-water calorifiers there are two steam-heated calorifiers; one for use with exhaust steam, and one for live steam, to come into operation automatically if the hot-water heating coils from the economiser fail. The automatic valve which opens and allows the steam to enter the steam calorifiers when the hot-water calorifiers fail, is worked on very much the same lines as that controlling the damper of the hot-water boilers. There is a tank on the roof containing a supply of cold water which has passed through a Boby's water-softening apparatus. In addition, there are four very large tanks under the basement collecting rain-water drained from the roofs.

The closed water circuit formed by the economisers and caliorifiers prevents the formation of deposit from hard water inside the economiser tubes, a trouble that has lessened the use of the economisers in some districts.

* The economiser was described in an article in the columns of THE HOSPITAL some months back.

## Figures and Tables

**Fig. 1. f1:**
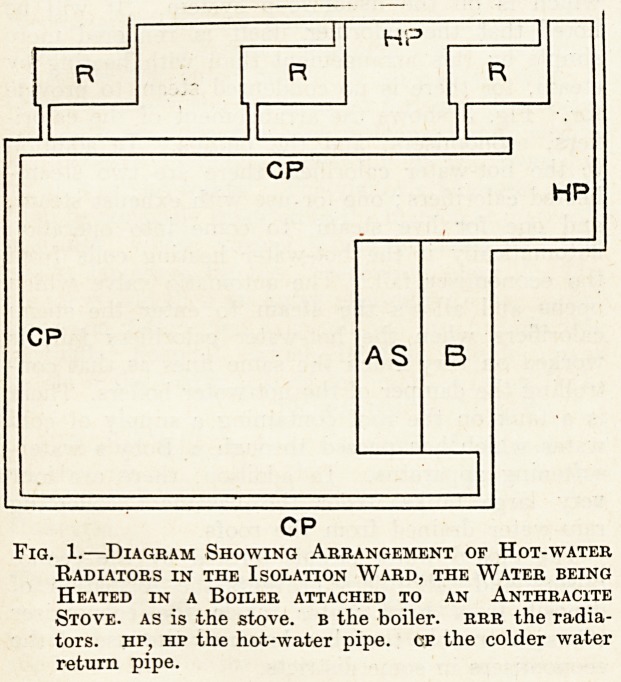


**Fig. 2. f2:**
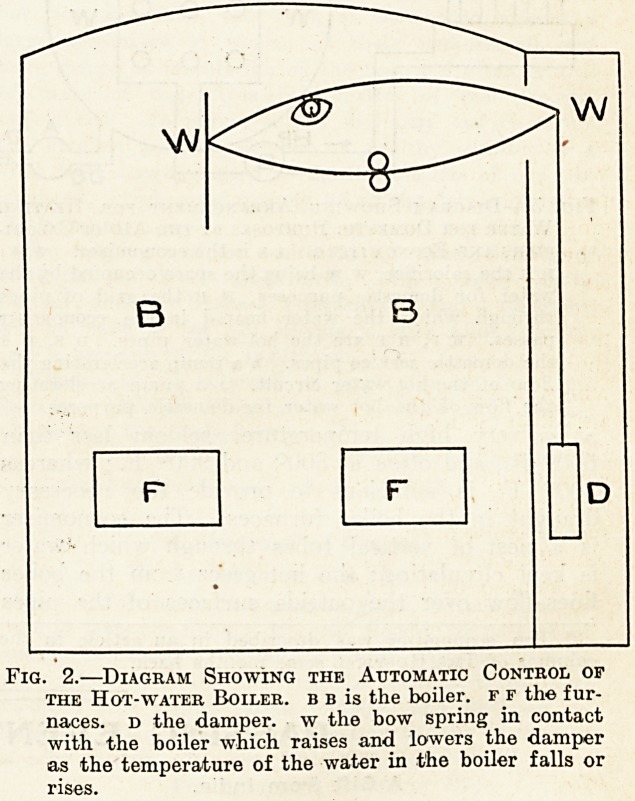


**Fig. 3. f3:**